# Mutations in *GHR* and *IGF1R* Genes as a Potential Reason for the Lack of Catch-Up Growth in SGA Children

**DOI:** 10.3390/genes13050856

**Published:** 2022-05-11

**Authors:** Weronika Stróżewska, Magdalena Durda-Masny, Anita Szwed

**Affiliations:** Institute of Human Biology and Evolution, Faculty of Biology, Adam Mickiewicz University, Uniwersytetu Poznańskiego 6, 61-614 Poznan, Poland; werstr1@amu.edu.pl (W.S.); mdurda@amu.edu.pl (M.D.-M.)

**Keywords:** growth hormone receptor, insulin-like growth factor 1 receptor, SGA, catch-up growth

## Abstract

The aim of this review was to describe all of the mutations in the growth hormone receptor (*GHR*) and insulin-like growth factor-1 receptor (*IGF1R*) genes that have been discovered so far, and their possible impact on final body height, as well as their relationship with catch-up growth in children born small for gestational age (SGA). Mutations in the *GHR* gene were found to cause a body height below −2 SD, from the mean for sex and age, whereas the mutations in the *IGF1R* gene were associated with low body height and intrauterine growth restriction (IUGR), and with being born SGA. After birth, when the child’s growth is not restricted by the intrauterine environment, the infant may develop its developmental potential and experience catch-up growth, which makes it possible to catch up with peers born appropriate for gestational age (AGA). Despite this, catch-up growth does not apply to all, but only to about 85% of SGA children, and its mechanism is unknown. It is possible that SGA children who did not experience catch-up growth are carriers of mutations in the *GHR* and/or *IGF1R* genes

## 1. Introduction

The birth size of a newborn child is influenced by a number of factors. The main ones are genetic factors of the fetus and the intrauterine environment. These factors interact with each other, and the effect of this interaction is seen as the birth weight, length, body composition, and organ size [1]. From conception to delivery, the fetus is under the influence of the mother’s organism, which is the environment for the developing organism. The capacity of the uterus corresponds to the mother’s height and it is one of the main determinants of the fetus’s size. In addition, the birth size of the fetus can be influenced by the nutritional status of the mother, which provides nutrients to developing organisms [2].

If the fetus is too large, this exposes the mother to complications that are related to childbirth. If the fetus is too small, this increases the risk of the newborn’s illness or death after birth, and it may increase the risk of disease in later life, such as obesity or cardiovascular disease [1]. The International Classification of Diseases (ICD 10) defines children born with a weight below 2500 g, regardless of their fetal age, as those born with low body weights [2]. The definition of children born small for gestational age (SGA) is also important. These newborns have reported body weights and/or lengths below the 10th percentile, or more than −2 SD below the mean of the children’s birth weights and/or lengths. About 11% of all children born annually in the world are defined as SGA, and 10% of them are below the 3rd percentile in terms of body height in adulthood [3]. Although SGA children are born with low body weights and/or heights, up to 85% of them experience catch-up growth within the first 2 years of life [4]. Catch-up growth means the speed of growth above the limits of the statistical norms for a given age and sex, which allows SGA children to catch up to their peers who were born with weights and/or lengths appropriate to their fetal ages [5].

**Figure 1:**
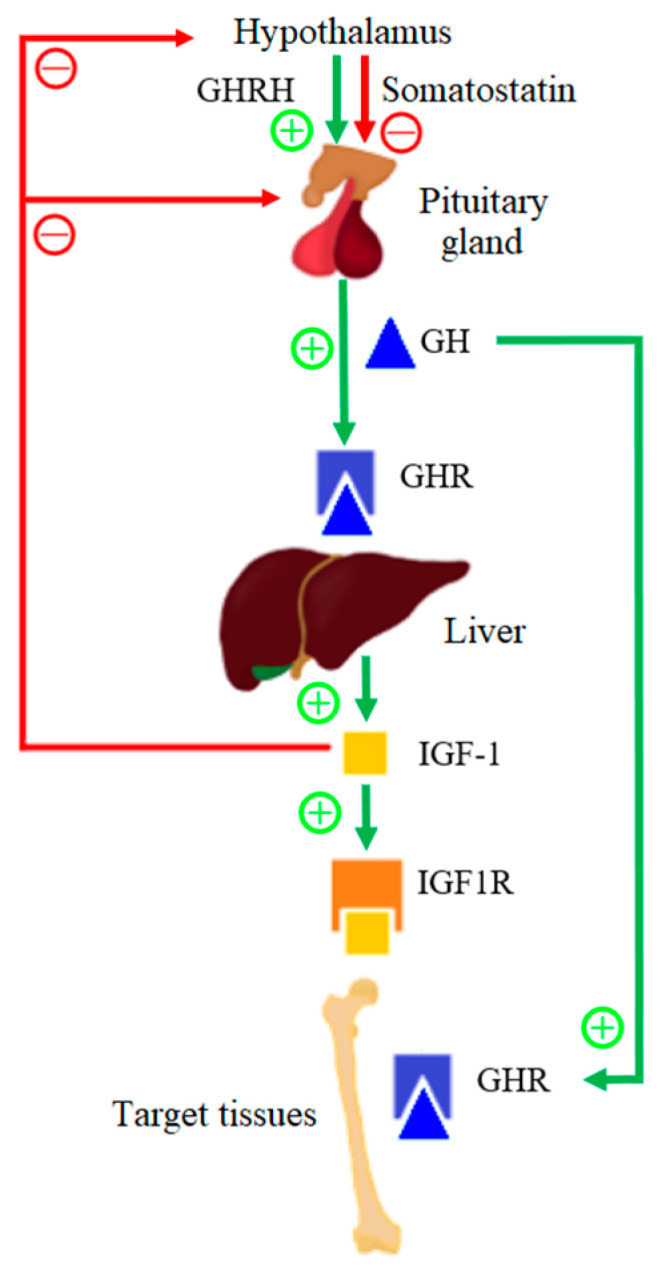
Functional diagram of the GH–IGF-1 axis.

Growth hormone (GH) and insulin-like growth factor 1 (IGF-1) play an important role in the growth process; however, GH deficiency is rare in SGA children [5]. Infant development is, first, a continuation of a fetal growth in which IGF-1 plays a dominant role; however, the growth rate slows down after the first year of life [6]. The GH–IGF-1 axis is the main endocrine growth regulator in children that are over 1 year old. The scheme of operation of the GH–IGF-1 axis is presented in Figure 1. Growth hormone-releasing hormone (GHRH) is secreted by pulsation by the hypothalamus and it positively influences the release of GH by the anterior pituitary gland. The hypothalamus also secretes somatostatin, which negatively affects the secretion of GH by the pituitary gland. About 50% of GH in serum is bound to the growth hormone-binding protein (GHBP), which corresponds to the extracellular domain of the GH receptor (GHR). GHBP increases GH in the serum half-life, and modulates the GH bioactivity by competing for GH with GHR [7]. After biologically active GH binds to its receptor, GHR dimerizes and, as a result, the intracellular signaling pathway is activated, which leads to the secretion of IGF-1 from various tissues, but mainly from liver tissues [8]. IGF-1 mediates the anabolic and mitogenic activity of GH [9].

IGF-1 is a ligand for the insulin-like growth factor-1 receptor (IGF1R). Up to 75% of serum IGF-1 produces a ternary complex that consists of IGF-1, insulin-like growth factor binding protein-3 (IGFBP-3), and an acid-labile subunit (ALS), and 25% form double complexes with only IGFBP. IGFBPs are produced in the liver under the influence of GH. After IGFBP binds itself to IGF-1, it prolongs the half-life of serum IGF-1. After the binding of IGF-1 to IGF1R, a signaling pathway is activated that causes cell proliferation and differentiation, and it therefore has a mitogenic effect [10]. Moreover, the GH–IGF-1 axis has been shown to be important in the postnatal longitudinal bone growth, as it is believed that GH and IGF-1 affect the parathyroid hormone-related protein (PTHrP). PTHrP is expressed in the population of the resting zone of chondrocytes, which are skeletal stem cells, and it is confirmed that GH has a direct effect on actively proliferating chondrocytes and, therefore, on bone growth.

Receptors play a key role in the function of the GH–IGF-1 axis. GHR regulates the IGF-1 expression; therefore, its proper structure is extremely important in the growth process. A dysfunctional GHR can lead to a defective GH response and can cause a low final height phenotype, but it can also lead to decreased bone-mineral density, obesity, a higher risk of osteoporosis, cardiovascular disease, and lipid disorders [11,12]. Mutations in the *IGF1R* gene may affect IGF-1 binding to IGF1R and/or a reduced number of extracellular receptors [13].

The clinical approach to the issue of the idiopathic short stature is changing with time. It is expected that the diagnostic methods of short stature will soon change. One of the new approaches should be genetic analysis [14]. This would provide the prediction of an assumed final body height at a very young age on the basis of the patient genotype. Wit et al. [15] have already proposed that children with IGF-1 deficiency should undergo genetic analysis. Children who have expected mutations that cause, for example, the limited secretion of IGF-1, could undergo hormonal therapy, which would help them to reach higher final body heights. However, to this day, there are no guidelines that specify which mutations should be searched for within short children or SGA children because it is not yet clear which mutations cause short stature. The mutations that are described in this review might be responsible for the lack of catch-up growth and, therefore, they could be good candidates for genetic testing for the counteraction of short stature.

Because of the premises concerning the influence of mutations in *GHR* and *IGF1R* genes on low birth height and final height, it is necessary to conduct clinical trials to confirm these suspicions. The aim of this review was to describe all of the mutations that cause partial insensitivity to GH in the *GHR* gene and those mutations that have been discovered in the *IGF1R* gene, as well as to present the impact of these mutations on the phenotype.

### 1.1. Growth Hormone Receptor

The *GHR* gene is located on the 5p13.1-p12 chromosome and it is approximately 87 kbp. *GHR* is encoded by nine exons, the coding region, and a 3’ untranslated region. Eight of these exons are between 66 and 179 bp in size, with the last one, which encodes almost the entire cytoplasmic domain, being approximately 3400 bp [16]. 

Two isoforms of *GHR* have been distinguished in humans. Full-length isoform (*fl-GHR*) and an isoform that is lacking exon 3 (*d3-GHR*). By comparing the two isoforms in vitro, after the exposure to GH, it was found that the *d3-GHR* polymorphism has 1,7 to 2,0 times faster signal transduction than the full-length isoform. An association has also been noted between *d3-GHR* and an increased response to GH therapy in SGA children [17,18]. The largest number of GHRs is found in the liver; however, they are also found in other tissues and organs, such as the growth plate adipose tissue, muscle, and kidneys [9].

The growth process is initiated by binding the growth hormone to the GHR, which activates the intracellular signaling path through dimerization. GHR-related intracellular Janus tyrosine kinase 2 (JAK2) and, among others, the transcription activator 5 (STAT5), affect the expression of the IGF-1 gene, which results in the secretion of IGF-1. After the IGF-1 binds itself to IGF1R, this complex activates a mitogenic and anabolic response in target cells that lead to growth [19]. The extracellular domain of the GHR corresponds to GHBP, which is complexed with about half of the GH in human plasma [7].

**Figure 2:**
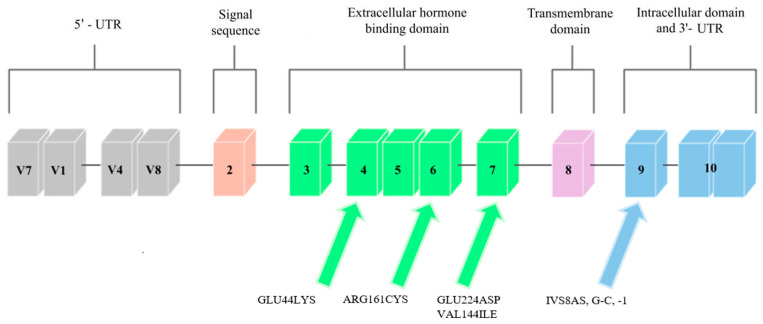
Scheme of the *GHR* gene. Arrows point to exons where mutations related to low body height or SGA/IUGR defined at birth have been found.

Mutations in the *GHR* gene cause growth hormone insensitivity syndrome (Laron’s syndrome), as well as partial growth hormone sensitivity [20]. As of today, 26 mutations that are responsible for Laron’s syndrome have been discovered: one that is responsible for hypercholesterolemia, one for increased responsiveness to GH, and five that are responsible for partial sensitivity to growth hormone. The latter, which are the aim of this study, are presented in Figure 2.

Three mutations that cause partial GH sensitivity in the *GHR* gene were discovered by Goddard et al. [21] in 4 out of 14 children with idiopathic short statures. The subjects were selected on the basis of the normal levels of growth-hormone secretion and the low serum levels of GH-binding protein.

Most of the previously identified mutations were located in the extracellular domain of the GHR, which, consequently, led to a decrease in the level of serum GHBP (which is derived from this receptor region). The results of the study by Goddard et al. [21] suggest that heterozygous mutations in the *GHR* gene may account for approximately 5% of idiopathic short-stature patients.

### 1.2. Insulin-like Growth Factor-1 Receptor

**Figure 3:**
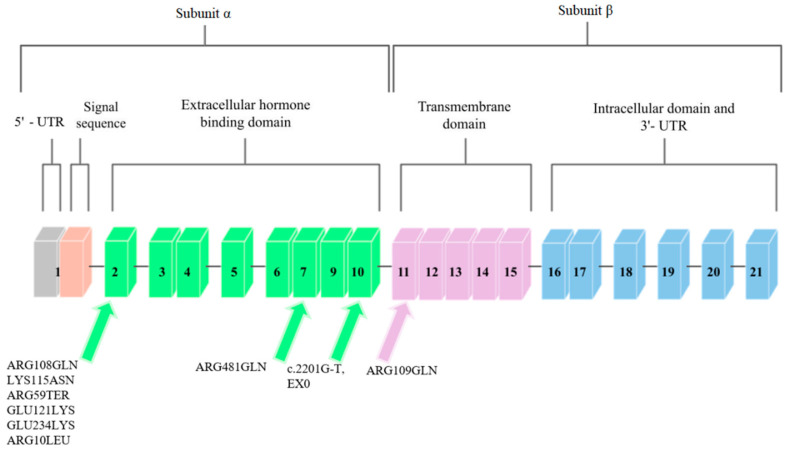
Schematic diagram of the *IGF1R* gene. Arrows point to exons where mutations related to low body height or SGA/IUGR defined at birth have been found.

The *IGF1R* gene is located on chromosome 15q.25–q.26 [22]. It covers approximately 100 kb, and the coding sequence of this gene is contained in 21 exons. Cooke et al. [23] analyzed the promoter region and found that the flanking and untranslated region is rich in guanine–cytosine pairs. The diagram of this gene is shown in Figure 3.

*IGF1R* is homologous to the insulin receptor gene with regard to amino acid sequences, which are more than 50% identical, and with regard to the organization of introns and exons. These genes code the precursor proteins that are post-translationally modified to produce receptors that consist of two α subunits that are extracellular and that contain ligand-binding domains and two β that contain intracellular tyrosine kinase domains. The complete absence of IGF-1 receptors may cause severe disease or may be lethal in humans, and less severe disorders, such as naturally occurring nonsense mutations, result in insulin resistance and IGF-1 resistance [24].

IGF1R shows a high level of homology with the insulin receptor, and it activates comparable signal transduction pathways. IGF1R can also form hybrid receptors with the insulin receptor [25]. Ligand-IGF1R binding leads to the autophosphorylation of this receptor and to tyrosine phosphorylation. Subsequently, substrate phosphorylation activates two major signaling pathways, which are PI3K/AKT/mTOR and Ras-MAPK [26].

Mutations in the *IGF1R* gene can confer IGF-1 resistance and can, hence, cause some cases of prenatal and postnatal growth restriction [24]. Nine mutations in the *IGF1R* gene have been described so far. They are presented in Figure 3.

A case of a person with a chromosome 15q26.1-qter deletion and monozygotic *IGF1R* gene was described. The patient exhibited Intrauterine Growth Restriction (IUGR), delayed growth and development, and, inter alia, microcephaly, lung underdevelopment, and kidney abnormalities. On the basis of other patients with similar chromosome 15 deletions, the study authors suggest that the absence of the *IGF1R* allele may be associated with the development of IUGR and postnatal growth deficiency [27].

### 1.3. Methods

Studies that describe the GHR and IGF1R and, in particular, the mutations that have been identified in the genes of these receptors, are the main part of this work. The mutations found in the *GHR* and *IGF1R* genes were searched for in the ClinVar and OMIM databases. Out of all 33 mutations described in the *GHR* gene, 28 mutations were excluded for this analysis, 26 of which were responsible for Laron’s syndrome, 1 of which caused hypercholesterolemia, and 1 of which caused increased responsiveness to GH. All of the other mutations that are responsible for the GHR partial intensity to GH are described. With regard to *IGF1R*, all mutations discovered so far have been described. The publications in which the mutations were reported were found by using the ClinVar database, in which the section “Citations for this variant” lists all of the publications in which this mutation is mentioned. Publications were obtained from the following databases: Pubmed, Scopus, and Web of Science. A search for additional literature was also carried out. The keywords were “GHR”, “IGF1R”, “mutations”, “GH”, “IGF1”, “SGA”, and “catch-up growth”. Table 1 shows a list of all of the publications that were used in which mutations in *GHR* and *IGF1R* were described, along with the characteristics of the methods and the material used in these publications.

### 1.4. Mutations in the GHR Gene 

#### 1.4.1. GLU44LYS, rs121909361

One of the mutations that was diagnosed by Goddard et al. [21] was a mutation located in exon 4 that introduced lysine instead of glutamic acid at position 44 (Glu44Lys) in 184 base pairs (Figure 2). The mutation in this exon was inherited from the father. The authors of the study analyzed the effect of this mutation on the GH-binding ability of GHR. The Glu44 residue is in direct contact with the GH, and the introduction of lysine at position 44 reduced the binding by 330, compared to the wild-type receptor binding. It was therefore found that this mutation is responsible for a decreased affinity for GH.

#### 1.4.2. ARG161CYS, rs121909362

Another mutation that was reported by Goddard et al. [21] was a mutation in exon 6, at 535 base pairs (Figure 2). It introduced a cysteine at position 161 (Arg161Cys) in place of arginine. The cysteine in this mutation was potentially misfolded. The mutation was inherited from the mother’s side. One of the patients studied by Goddard was heterozygously composed of the Glu44Lys and Arg161Cys mutations, and he was more affected by partial GH sensitivity than his homozygous parents (father had the Glu44Lys mutation, mother had Arg161Cys). Similar to the rest of the study participants, he was treated with GH, but the response to this treatment was poor, and so it was concluded that the reason for this may be mutations that cause partial sensitivity to GH due to dysfunctional GHRs.

#### 1.4.3. GLU224ASP, rs45588036

The last mutation discovered by Goddard et al. [21] was found in exon 7 (Figure 2), and it was a mutation in the 726 base pair that introduced aspartic acid in place of glutamic acid at position 224 (Glu224Asp). However, the conservative substitution in this mutation was expressed at normal expression levels and resembled the mutant Arg211His protein, so that the GH affinity for the *GHR* gene that contains the Glu224Asp mutation was nearly normal. A possible effect of this mutation was incorrect subcellular localization. Partial GH insensitivity, which can be manifested by short stature, may occur in individuals who have heterozygous mutations in the *GHR* gene due to mutations in other genes, or when changes confer a dominant phenotype. Patients were selected, in part, on the basis of decreased serum GHBP levels, which suggest that the mutations discussed by Goddard may directly affect ligand binding. In the case of the Glu44Lys mutation, a 330-fold decrease in the affinity of the GH receptor to GHBP was observed, while the remaining mutations had smaller effects on the ligand binding.

#### 1.4.4. IVS8AS, G-C, -1 rs730880308

Ayling et al. [28] report the case of a family in which a GHIS causal mutation, IVS8AS, was recognized in the mother and daughter, which was located in the cytoplasmic domain of the GH receptor, which is an inherited domain. It was a single heterozygous mutation that transverses G to C at position 1 of the 3′ splicing acceptor site that precedes exon 9 of the *GHR* (Figure 2). The fusion of JAK2 with the GHR requires a conserved exon 9-encoded sequence that is located in the cytoplasmic domain. This mutation had a dominant negative effect, which caused a shortening of the cytoplasmic part of the protein, and it was associated with GHBP. Most of the previously discovered *GHR* mutations were located in the extracellular binding domain of the GHR. However, in the study, Ayling indicates that dominant *GHR* mutations should be looked for in children with normal GHBP levels and low body heights; that is, in children with no previous suspected endocrinopathy.

#### 1.4.5. VAL144ILE, rs6413484

A study by Sanchez et al. [13] discovered the last known mutation in the growth hormone receptor gene so far, which was the VAL144ILE mutation, which is located on exon 6. It was found in one subject with a short body height and in two members of her family, who also had shorter body heights (Figure 2). A study by Amslem et al. [20] describes the same amino acid that is changed in this family. The significance of this mutation has not been fully established; however, it seems likely that this mutation affects height. The potential significance of this mutation was established from the above-described work by Ayling et al. [28], in which the patient with GHIS had a mutated allele on the same amino acid (144) as the patient in the study by Sanchez et al. [13]. However, this mutation occurred in a different base pair. This change was related to GHIS, and so the amino acid V144 was found to be important for the GH receptor function.

### 1.5. Mutations in the IGF1R Gene 

#### 1.5.1. ARG108GLN, rs121912426

Abuzzahab et al. [24], after examining 51 people who showed short postnatal growth, found a mutation in the *IGF1R* gene in two of them. Both of these mutations were found in exon 2 (Figure 3). The first person described had two mutations. The first was a single base pair substitution at the codon of amino acid 108 that changed arginine into glutamine. The mutation at position 108, ARG108GLN, was inherited from the father. This mutation affected the α and β cleavage site, which resulted in the intracellular sequestration of the unprocessed proreceptor, and decreased *IGF1R* expression [32].

#### 1.5.2. LYS115ASN, rs121912427

The second mutation described by Abuzzahab et al. [24] in the first of 51 patients was a mutation in *IGF1R* that consisted of replacing lysine with asparagine at amino acid codon 115 (Figure 3). This mutation, LYS115ASN, was inherited from the mother. In the person who had these mutations, each of the α subunits in the mature receptor contained one of these mutations in various combinations, which is due to the heterotetrameric structure of IGF1R. These mutations changed the conformation of the IGF1R-binding domain and, consequently, reduced ligand binding. The binding of IGF-1 to IGF1R on the fibroblasts in the patient was reduced by one-third compared to the control group. Receptor phosphorylation in response to IGF-1 was also investigated to determine decreased hormone binding to receptor signaling. Phosphorylation was dose-dependent in fibroblasts; however, it showed a decrease in sensitivity compared to the control group. The subject was treated with growth hormone, during which the concentration of IGF-1 in the serum increased, which indicated abnormal GH secretion.

#### 1.5.3. ARG59TER, rs121912428

Abuzzahab et al. [24] also found mutations in a patient who showed a low body height and a slow growth rate. This patient was heterozygous for the early transcriptional termination mutation at exon 2 at position 59, which should contain arginine (Figure 3). This mutation was inherited from the mother, who was also born SGA. This mutation inactivates one *IGF1R* allele, and the protein may not be expressed; however, if expressed, it would only correspond to the N-terminal end of the *IGF1R* α chain, and the receptor would not be able to bind IGF-1. It was suggested that individuals with the ARG59TER mutation may exhibit a haploinsufficiency phenotype, as the biallelic expression of wild-type and mutant *IGF1R* alleles was detected in the blood of the patient. After examining the phosphorylation of Akt, the *IGF1R* signaling protein, and following an analysis of the post-receptor signal transduction, it was proposed that the IGF-1 resistance in the subject was due to a reduced number of IGF1R [32].

#### 1.5.4. ARG709GLN, rs121012429

The ARG709GLN mutation was discovered by Kawashima et al. [29]. They examined 24 people who were characterized by low body heights and IUGR. One person was identified with a mutation in the *IGF1R* gene that was heterozygous for arginine to glutamine conversion at position 709 (Figure 3). This mutation was inherited from the mother and it changed the IGF1R proreceptor cleavage site by losing the restriction site. ARG709GLN affected the cleavage site between the α and β subunits, which caused the intracellular sequestration of the unprocessed proreceptor, and the decreased expression of fully digested and processed IGF1R [32].

#### 1.5.5. ARG481GLN, rs33958176

In another study, Inagaki et al. [30] describes a mutation that was found in a girl with a short body height. This mutation was based on the replacement of arginine with glutamine at position 481 of exon 7 *IGF1R* (Figure 3). This mutation was inherited from the maternal side. Researchers observed an increase in tyrosine phosphorylation after IGF-1 stimulation in the mutant *IGF1R,* compared to wild-type, and decreases in Akt phosphorylation and in proliferation in cells that overexpressed mutant *IGF1R*. Because of the mutation site being close to the first of the four disulfide bonds that connect the α and β subunits, it was suggested that this mutation disrupted this binding and resulted in incomplete dimerization and conformational alteration.

#### 1.5.6. GLU121LYS, rs1555434208

The GLU121LYS and GLU234LYS mutations were discovered and described in the study by Fang et al. [31]. Two siblings were examined, one of whom had IUGR, and the other who had a short body height. Subjects had complex heterozygous missense mutations in the *IGF1R* gene. One was found in exon 2 and consisted of replacing glutamic acid with lysine at position 121 (Figure 3). This mutation was also found in the mother of the studied siblings.

#### 1.5.7. GLU234LYS, rs1253103806

The second mutation reported by Fang et al. [31] is the mutation that is involved in replacing glutamic acid with lysine at position 234 of exon 2 (Figure 3). This mutation was inherited from the father of the subjects. These mutations resulted in decreased expression of mature and functional IGF1R peptides, with a consequently decreased activation of the IGF1R pathway in response to IGF-1. Analysis of sibling fibroblasts showed an 80% reduction in processed α and β subunits, compared to wild-type receptors.

#### 1.5.8. ARG10LEU, rs1409058783

The penultimate mutation identified so far in the *IGF1R* gene was discovered and described by Gannage-Yared et al. [25]. They examined a patient born with IUGR who exhibited low body height later in life. A missense mutation in exon 2 was identified to convert arginine to leucine at position 10 (Figure 3). This mutation was inherited from both parents, who were its heterozygous carriers. This mutation resulted in the loss of hydrogen bonds between Leu10 and Asp8 in the *IGF1R*. The total expression of *IGF1R* containing this mutation was unchanged; however, impaired IGF receptor activation and decreased IGF1R autophosphorylation were observed in the subject’s fibroblasts, and greater amounts of IGF-1 were required to achieve a similar level of Akt phosphorylation as in the controls with receptors without this mutation.

#### 1.5.9. EX10, rs1555460945, c.2201G-T

The most recent mutation in the *IGF1R* gene to date is reported by Prontera et al. [26]. The mutation carrier was a patient born with IUGR. The mutation in this person consisted of a c.2201G-T transversion that was identified on the last nucleotide in exon 10 (Figure 3). The examined person showed reduced autophosphorylation in fibroblasts and lower Akt activity, and an abnormal isoform that generates a mutant protein that contains 25 additional amino acids. The studies carried out by the authors of the study indicate the impaired ability of the *IGF1R* that contains this mutation to self-phosphorylate and to activate the downstream IGF-1 signaling pathway and the insulin–Akt complex.

## 2. Conclusions

It has not yet been established why some SGA children do not experience catch-up growth. In light of the increasing birth rate of SGA babies, it is important to intensify research into finding the causes of persistent low body heights in some of them. It seems that mutations in the *IGF1R* gene, and mutations that cause partial insensitivity to the growth hormone in the *GHR* gene that are presented in the review may be related to the lack of catch-up growth in a part of SGA children. Proving the existence of this relationship in clinical trials, and then introducing genetic analysis as a standard procedure in the case of SGA children, would significantly help in making decisions about possible treatment modalities.

## Figures and Tables

**Table 1:** List of publications that describe mutations in the *GHR* and the *IGF1R* genes, along with the number of people tested in these studies, the discovered mutations, and a description of the qualification criteria for the tests.

Author, Year	Mutation	Number of Tested Persons	Qualifications for Research
Goddard et al., 1995 [21]	GLU44LYS, rs121909361; ARG161CYS, rs121909362; GLU224ASP, rs45588036	14	Low body height; more than 2,5 SD below the mean; SD of the serum GHBP concentration less than −2 SD from the mean for age and sex; SD results for serum IGF-I concentration less than 0 for age and sex; maximum GH concentration in serum levels above 10 mg per liter; no systemic disease.
Ayling et al., 1997 [28]	IVS8AS, C-C, rs730880308	1	Low body height
Sanchez et al., 1998 [13]	VAL144ILE, rs6413484	17	Low body height; more than −2 SD below mean; normal serum GH level; normal IGF-1 level; children with known causes of low height were excluded.
Abuzzahab et al., 2003 [24]	ARG108GLN, rs121912426; LYS115ASN, rs121912427	42	Children with IUGR; low body height (less than −2 SD after 18 months of age); children with serum IGF-1 and IGFBP-3 below the normal reference range for age were excluded; chronically ill children.
Abuzzahab et al., 2003 [24]	ARG59TER, rs121912428	50	Children with potential IGF-1 resistance who had body heights more than −2,5 SD below the mean for a given age; a serum IGF-1 concentration more than 2 SD above the mean for age and sex.
Kawashima et al., 2005 [29]	ARG709GLN, rs121012429	24	Low body height; more than −2 SD below the mean; IUGR defined at birth.
Inagaki et al., 2007 [30]	ARG481GLN, rs33958176	1	Low body height; more than −5 SD below the mean SGA.
Fang et al., 2012 [31]	GLU121LYS, rs1555434208; GLU234LYS, rs1253103806	2	Low body height; more than −3 SD below the mean; IUGR and SGA defined at birth.
Gannage-Yared et al., 2013 [31]	ARG10LEU, rs1409058783	1	Low body height; more than −3 SD below the mean; IUGR defined at birth.
Prontera et al., 2015 [26]	EX10, rs1555460945, c.2201G-T	1	Low body height; more than −3 SD below the mean; IUGR defined at birth.
